# The impact of health insurance on rural energy transition: evidence from rural China

**DOI:** 10.3389/fpubh.2026.1752398

**Published:** 2026-07-06

**Authors:** Hao Wang, Zhina Wang

**Affiliations:** School of Business, Xinyang Normal University, Xinyang, China

**Keywords:** health insurance, medical resource endowment, rural households, social security, sustainable energy transformation

## Abstract

**Introduction:**

Although many studies in developing countries have paid attention to the importance of rural energy transition, the significance of the healthcare system in the energy transition process has not received sufficient attention. This study investigates whether and how participation in the New Rural Cooperative Medical Scheme (NRCMS) affects rural household clean energy consumption.

**Methods:**

Using panel data from the China Family Panel Studies (CFPS) for 2018 and 2020, covering 8,427 rural households across 29 provinces, we employ fixed- effects and instrumental variable models to address potential endogeneity.

**Results:**

The results show that NRCMS participation significantly increases the probability of adopting clean energy by approximately 2.8 percentage points. Mechanism analysis indicates that the effect operates primarily through wealth effect (reducing medical burden) and the health effect, while the precautionary savings channel is not supported. The heterogeneity analysis shows that households with low expenditure pressure are more likely to be incentivized by the NRCMS to consume clean energy than those with a larger number of unmarried members. Similarly, households with a smaller share of migrant workers are more likely to increase clean energy consumption after participating in the NRCMS than those with a larger share. Furthermore, for every one unit increase in regional medical resource endowment, the promoting effect of the NRCMS participation on rural household clean energy consumption increases by 0. 016 units.

**Discussion:**

These findings provide new insights for improving the rural medical insurance system and accelerating the transformation of rural energy consumption.

## 1 Introduction

Ensuring that everyone has access to affordable, reliable, sustainable, and modern energy has been listed as one of the Sustainable Development Goals (SDGs) by the United Nations in 2015. Long-term consumption of non-clean energy not only intensifies adverse ecological impacts, exemplified by deforestation and air quality deterioration (1), but also elevates the risk of health issues among rural populations, including tuberculosis, lung cancer, and respiratory infections (2–4). However, widespread reliance on traditional energy sources such as fuelwood, straw and coal continues to be prevalent in rural households in many developing countries (5). According to data released by the IEA, there are about 2.5 billion people relying on traditional solid energy sources as their main source cooking energy in 2020 worldwide. As the largest developing country, the energy use structure of most rural households in China is still characterized by solid energy as the main source of cooking energy (3). According to data from the Third National Agricultural Census, there are still 68.1% of rural households in China that use traditional energy sources as their main source of energy consumption. Furthermore, the rich food culture in China has driven a much higher proportion of traditional solid energy consumption than in other developing countries (6). The reliance on traditional solid energy sources in many rural households hinders the achievement of the SDGs and poses substantial health and environmental risks. The transition to clean energy is crucial for mitigating negative ecological impacts, reducing the prevalence of diseases among rural residents, and improving overall quality of life. Given China’s status as the largest developing country, addressing this issue is not only important for sustainable development in China, but also has global implications for the world’s sustainable development.

## 2 Literature review

### 2.1 Determinants of rural household clean energy transition

Under the analytical framework of neoclassical economics, farmers are regarded as economically rational individuals who can make effective judgments on costs and benefits and adjust their use of factors of production to maximize returns (7, 8). Because biomass energy is more readily available in rural areas with lower collection costs, rural household energy use has long been dominated by biomass sources such as fuelwood and straw. In contrast, piped gas, bottled liquefied petroleum gas, and electrical appliances are more widely used in urban households (9). With improvements in household economic conditions, farmers’ energy choices have gradually shifted from passive adaptation to active pursuit, emphasizing convenience, safety, and cleanliness (10). In addition, accessing low-cost non-clean energy requires labor inputs. When household labor shifts to off-farm employment, the opportunity cost of using non-clean energy increases significantly, making farmers more inclined to use clean energy (11).

Furthermore, the analytical framework of energy consumption choices has incorporated psychological factors such as farmers’ perceived expected returns, health awareness, and environmental awareness (12, 13). Specifically, farmers’ clean energy consumption is affected by expected uncertainty. Because market economies are inherently risky and unpredictable health shocks may affect household welfare, farmers feel more confident in using clean energy when they believe their income will remain stable (14). Stronger environmental and health awareness significantly influences rural households’ energy improvement investment decisions, enhancing their perceptions of clean energy and motivating them to improve well-being (15). In summary, existing literature suggests that the transition to clean energy consumption in rural households requires both economic and psychological factors.

This observation raises an important question: can public policies that improve household economic security and health conditions indirectly promote clean energy consumption? Health insurance provides a particularly relevant context for addressing this issue.

### 2.2 Economic and behavioral consequences of health insurance

The New Rural Cooperative Medical Scheme (NRCMS) was piloted in rural China in 2003 and has made important contributions to reducing the medical burden of rural families and improving residents’ health status (16–18). Studies on the NRCMS can be classified into two categories. The first examines its positive effects, particularly its push-pull effect on labor mobility (19). The second focuses on negative effects arising from the voluntary participation nature of the NRCMS, which inevitably leads to adverse selection and the negative externality of wasted medical resources (20–22). More recently, scholars have begun to examine the non-medical spillover effects of the NRCMS, such as its impact on non-medical household consumption (e.g., durable goods, education expenditure) (23, 24). However, little attention has been paid to its relationship with household energy consumption. However, whether these economic and behavioral changes translate into cleaner household energy choices remains largely unexplored.

### 2.3 Health insurance and household consumption behavior

A key channel through which health insurance may influence household decisions is consumption behavior. Existing studies suggest that health insurance affects household consumption through both wealth-related and health-related mechanisms (25, 26). From a wealth perspective, health insurance reduces out-of-pocket medical expenditures and lowers future health-related financial uncertainty, thereby relaxing household budget constraints and potentially increasing consumption of higher-quality goods and services (27). From a health perspective, improved health status and enhanced health awareness may reshape household preferences toward products and behaviors that contribute to healthier living environments. Importantly, these mechanisms are closely related to the determinants of clean energy adoption identified in the energy-transition literature. Clean energy consumption often requires greater financial resources than traditional fuels, while health concerns associated with indoor air pollution constitute an important motivation for fuel switching. Therefore, the wealth and health effects generated by health insurance may provide a previously overlooked pathway linking social protection policies to rural household energy transition.

Despite these theoretical connections, direct empirical evidence on whether and how health insurance affects rural household clean energy consumption remains scarce. This gap motivates the present study.

### 2.4 Research gap

Although the existing literature provides important insights into household energy transition, health insurance effects, and household consumption behavior, several gaps remain.

First, studies on rural household clean energy consumption primarily focus on socioeconomic characteristics, infrastructure conditions, and environmental awareness, while relatively little attention has been paid to the role of social protection policies. As a result, the potential contribution of health insurance programs to household energy transition remains insufficiently understood. Second, a large body of research has examined the effects of the NRCMS on medical expenditures, healthcare utilization, health outcomes, savings, and consumption. However, most studies concentrate on traditional economic and welfare outcomes and rarely explore whether health insurance can influence environmental or energy-related behaviors. Third, although previous studies have shown that health status and health awareness may affect household energy choices, the potential linkage between health insurance participation and clean energy adoption through wealth and health channels remains largely unexplored. To address these gaps, this study investigates whether and how participation in the NRCMS influences rural household clean energy consumption. By integrating perspectives from health economics and energy economics, this study contributes to a deeper understanding of the broader welfare and environmental implications of social health insurance programs.

## 3 Analysis framework

The New Rural Cooperative Medical Scheme (NRCMS)^1^ represents a quasi-natural experimental institutional arrangement that reshapes the intertemporal budget constraints and risk expectations of rural households. As a large-scale public health insurance program targeting rural residents, the NRCMS not only reduces ex-post medical expenditures but also fundamentally alters households’ ex-ante risk perceptions and their behavioral responses to uncertainty. These characteristics make it a highly valuable institutional setting for examining how social protection policies influence household consumption structures beyond healthcare, including energy consumption.

Household energy consumption—particularly the choice between traditional biomass energy and modern clean energy—is influenced by both liquidity constraints and risk considerations (28). Adopting clean energy typically requires higher upfront or recurrent expenditures, whereas traditional energy sources (e.g., firewood and coal) are relatively cheaper but more polluting. Therefore, understanding the transition toward clean energy necessitates embedding energy decisions within a broader framework that encompasses income allocation, risk management, and health awareness.

In this context, the NRCMS affects rural household energy consumption through two key channels: the wealth effect and the health effect. Importantly, these two mechanisms are conceptually different and operate under different conditions. The wealth effect works through the relaxation of household budget constraints. By reducing expected medical expenditures and lowering the financial risks associated with health shocks, the NRCMS increases disposable resources available for non-medical consumption. When rural households face liquidity constraints and clean energy involves higher monetary costs than traditional fuels, this relaxation of budget constraints increases the likelihood of adopting clean energy.

In contrast, the health effect operates through preference formation and behavioral responses rather than through financial resources. Participation in the NRCMS improves access to healthcare services, increases exposure to health-related information, and strengthens awareness of health risks. As households become more aware of the adverse health consequences associated with traditional fuels, they are more likely to develop preferences for cleaner and healthier energy sources.

Therefore, the wealth effect primarily operates through household budget constraints, whereas the health effect operates through changes in preferences, cognition, and behavioral decision-making. The two channels are theoretically distinct and complementary.

### 3.1 Wealth effects of the NRCMS and clean energy consumption

The wealth effect induced by participation in the NRCMS operates through two primary mechanisms. First, the NRCMS reduces out-of-pocket medical expenditures, thereby relaxing households’ budget constraints. By reimbursing a share of healthcare costs—including medication, diagnostics, and hospitalization—the program lowers the effective cost of illness and mitigates the burden of health shocks. This reduction in medical expenditure functions as an income-equivalent transfer (26), increasing disposable income available for other types of consumption. Given that clean energy technologies typically involve higher monetary costs than traditional fuels, improved liquidity conditions may facilitate households’ transition toward cleaner energy sources. Second, the NRCMS reduces precautionary savings by mitigating uncertainty associated with future medical expenses. According to the life-cycle/permanent income framework, households facing income volatility and expenditure uncertainty tend to accumulate buffer-stock savings at the expense of current consumption (27, 29). In rural China, where health shocks are a major source of financial risk, the historical absence of insurance led households to adopt conservative consumption strategies, including reliance on low-cost but highly polluting energy sources. By pooling medical risks and providing partial insurance against health-related shocks, the NRCMS lowers uncertainty and weakens the precautionary saving motive. As a result, households are more likely to reallocate resources toward current consumption, including investments in cleaner but more expensive energy. This mechanism highlights that energy consumption decisions are not isolated choices but are embedded within a broader portfolio of risk-coping strategies.

The wealth effect generated by the NRCMS may operate through two distinct but related channels: alleviating medical burdens and reducing precautionary savings. Although both channels ultimately relax household budget constraints, they differ in their underlying mechanisms.

The medical-burden channel primarily reflects a current-income effect. Participation in the NRCMS reduces out-of-pocket medical expenditures incurred by illness and treatment, thereby directly increasing disposable household resources available for other consumption purposes. Given that clean energy often requires higher upfront costs than traditional fuels, the reduction in actual medical expenditures may increase households’ ability to adopt cleaner energy sources. In contrast, the precautionary-saving channel reflects a future-risk effect. Rural households in China face substantial uncertainty regarding future health expenditures due to income instability, imperfect social security coverage, and limited access to high-quality healthcare resources. Under such circumstances, households often accumulate precautionary savings to cope with potential health shocks. By reducing uncertainty regarding future medical expenditures, the NRCMS may weaken the incentive to hold precautionary savings and release resources for current consumption, including clean energy investments.

Importantly, these two channels may operate differently in rural China. Due to strong intergenerational obligations and the prevalence of housing, education, and marriage-related expenditures, reductions in medical risk do not necessarily translate into lower precautionary savings. Consequently, the medical-burden channel may generate a more direct effect on household energy choices than the precautionary-saving channel.

Based on the above theoretical analysis, we propose the following research hypotheses.

*H1:* Participation in the NRCMS significantly promotes rural households’ clean energy consumption.

*H2:* Participation in the NRCMS promotes rural households’ clean energy consumption through the wealth effect.

### 3.2 Health effects of the NRCMS and clean energy consumption

In addition to the wealth effect, the NRCMS may influence rural household clean energy consumption through a health effect. Unlike the wealth effect, which primarily operates through household budget constraints, the health effect works by changing households’ health status and health-related perceptions.

First, the NRCMS may operate through a health-improvement pathway. By improving access to healthcare services and increasing healthcare utilization, the NRCMS contributes to better health outcomes among rural residents. Improved health status may increase households’ concern for environmental quality and long-term health benefits, thereby encouraging a transition away from traditional solid fuels toward cleaner energy sources. In this sense, improvements in health capital may reshape household preferences for health-related consumption (30). Second, the NRCMS may operate through a health-cognition pathway. Participation in healthcare services often exposes individuals to health consultations, health education, and public health information (31). These experiences may enhance awareness of the adverse health consequences associated with traditional fuels and indoor air pollution. As households become more aware of these health risks, they may be more willing to adopt cleaner energy sources. Importantly, the two pathways are likely to interact with each other. Improved health status may strengthen preferences for healthier lifestyles, while enhanced health cognition may facilitate the translation of such preferences into actual energy choices. Therefore, health improvement and health cognition jointly constitute the health-effect channel through which the NRCMS influences rural household clean energy consumption (25, 32).

Based on the above theoretical analysis, we propose the following research hypotheses.

*H3:* Participation in the NRCMS promotes rural households’ clean energy consumption through the health effect.

## 4 Data and methodology

### 4.1 Data

**Figure 1:**
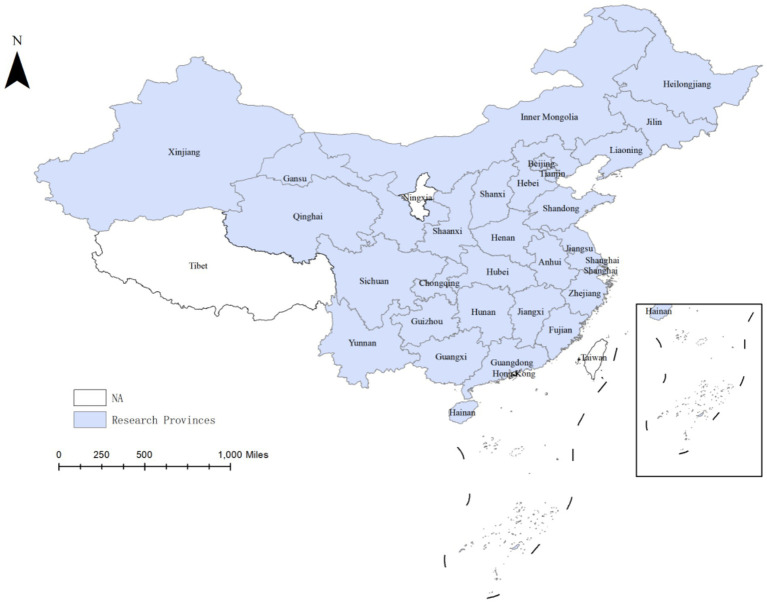
Geographic location of China’s provinces. NA means data is not available.

The dataset used in this study consist of two parts: we first select rural households from the CFPS dataset, then merge waves 2018 and 2020. Observations with missing values in key variables (energy choice, NRCMS participation, and controls) are excluded. The final sample includes 8,427 households across 742 villages. Given the national representativeness of CFPS^2^, the sample can reasonably reflect rural household characteristics in China. Our empirical analysis uses micro-data from 29 provinces of China. Figure 1 displays the geographic location of China’s provinces.

The second main dataset is the China Statistic Yearbook (2018, 2020). The control variables in this study include indicators such as the level of regional economic development, and the relevant data are collected from the China Statistical Yearbook^3^.

### 4.2 Variables and descriptive statistics

#### 4.2.1 Clean energy consumption

The explanatory variable in this paper is clean energy consumption, which is derived from responses to the CFPS household questionnaire, “What type of fuel does your household use most for cooking?”. Drawing on related studies (11, 33), households using clean energy sources (electricity, natural gas, gas, liquefied petroleum gas, solar energy, etc.) are assigned a value of 1, and households using non-clean energy sources (fuelwood, coal, etc.) are assigned a value of 0.

It should be noted that this study focuses on households’ primary energy choices rather than their complete energy consumption structure. The CFPS only records the primary cooking fuel used by households and does not provide detailed information on the quantity, proportion, or composition of different energy sources consumed. Consequently, it is not possible to fully capture fuel-stacking behavior among rural households. Nevertheless, primary cooking fuel remains one of the most widely used indicators in the household energy transition literature and serves as a meaningful proxy for the overall direction of household energy transition.

#### 4.2.2 The NRCMS

The dummy variable for whether or not a household participates in the NRCMS is used to indicate that, according to the NRCMS participation standard, farmers must participate in the NRCMS on a household basis, so the questionnaire’s question of whether or not to participate in the NRCMS reflects whether or not a household participates in the NRCMS, and specifically assigns a value of 1 to the families who are participating in the NRCMS, and 0 for vice versa.

#### 4.2.3 Control variables

To reduce the potential interference with the estimation results due to the omission of observable factors, we include a vector of exogenous factors as control variables. Specifically, individual characteristics mainly include gender, age, education, health, business insurance and pension insurance. Household characteristics mainly include income, family size, dependency ratio, fuel expenditure, and fixed assets. Regional characteristics mainly include regional economy, and Number of hospitals. Table 1 provides a statistical description of those variables.

**Table 1:** Variable definitions and descriptive statistics.

			Full sample		Sample of 2018		Sample of 2020	
Variable types	Variable	Definition and assignment	Mean	SD	Mean	SD	Mean	SD
Dependent variable	Energy	Whether to use clean fuels for cooking (0 = no; 1 = yes)	0.581	0.493	0.543	0.498	0.632	0.482
Core independent variable	NRCMS	Whether to participate in the NRCMS (0 = no; 1 = yes)	0.795	0.403	0.853	0.354	0.719	0.449
Control variables	Gender	Gender of the respondent (0 = female;1 = male)	0.546	0.498	0.557	0.497	0.532	0.499
	Age	Age of respondent in years	50.666	15.636	52.021	13.718	48.884	17.694
	Age^2^	Age square of the householder	2811.552	1569.405	2894.335	1416.333	2702.649	1744.713
	Education	Schooling of respondent (1 = illiteracy, 2 = elementary school, 3 = middle school, 4 = high school, 5 = post-secondary; 6 = university, 7 = graduate school; 8 = above)	2.360	1.172	2.259	1.102	2.492	1.246
	Health	Respondent' self-rated health (1 = very healthy, 2 = relatively healthy, 3 = average, 4 = relatively unhealthy, 5 = very unhealthy)	3.127	1.292	3.186	1.283	3.050	1.300
	Income	Total household income last year (take the logarithm plus one)	10.528	1.190	10.432	1.155	10.655	1.224
	Family size	Total number of members in a household	4.651	1.976	3.959	1.859	5.061	1.743
	Fuel expenditure	Household energy consumption expenditure over the past year (take the logarithm plus one)	3.416	2.081	3.384	2.067	3.459	2.098
	fixed assets	Total productive fixed assets of households (take the logarithm plus one)	2.746	3.080	2.186	1.241	3.494	4.371
	Pension insurance	Whether participating in pension insurance (0 = no; 1 = yes)	0.334	0.472	0.373	0.484	0.279	0.448
	Business insurance	Whether participating in business insurance (0 = no; 1 = yes)	0.254	0.435	0.240	0.427	0.273	0.445
	Dependency ratio	The ratio of households under 14 years old and over 64 years old (%)	0.638	0.312	0.477	0.323	0.849	0.095
	Regional economy	Regional GDP per capita (take the logarithm)	10.876	0.327	10.830	0.327	10.936	0.317
	Nh	Number of hospitals in districts (take the logarithm)	7.195	0.497	7.152	0.497	7.252	0.491
Instrumental Variable	Urban participation rate	The ratio of urban workers’ basic medical insurance enrolment to total population (%)	0.773	0.162	0.781	0.158	0.761	0.165

All empirical results are obtained based on data from the China Family Panel Studies (CFPS) 2018 and 2020 and the China Statistical Yearbook (2018 and 2020).

### 4.3 Methodology

#### 4.3.1 Estimation methods

Since the explanatory variables in this paper are binary discrete variables, the existing literature often uses probit or logit models. However, according to Angrist (34), the estimation of non-linear models requires more stringent restrictions on the distribution of the variables, the estimated coefficients are not averaging the treatment effects, and such models also suffer from conceptual ambiguities and other problems. In contrast, the traditional linear model, does not have these problems, therefore Angrist (34) argues that in applied research, the nonlinear model is not much superior to the traditional linear model in a comprehensive way. What’s more, when the endogenous variables are binary, traditional linear models tend to be easier to identify causal effects than nonlinear models without reliability. So, from the perspective of causal identification, ordinary least squares (OLS) is superior to probit or logit models in terms of endogeneity treatment, and that even when OLS estimation is used, the resulting bias is at most extrapolation bias rather than endogeneity bias, which is far more intolerable to the researcher than the former. To obtain the unbiased estimator of family cooking fuel selection for participating in the NRCMS. Referring to the study of Zheng (11), a multi-dimensional fixed-effects model is used to relieve the endogeneity problem due to the neglect of observable and unobservable factors. The model is as follows:

$$\text{Energ}y_{i}=\beta_{0}+\beta_{1}{\text{NRCMS}}_{i}+\text{θcontro}l_{i}+\gamma_{i}+\sigma_{t}+\varepsilon_{\mathrm{it}}$$ (1)

Where the dependent variable is $\text{Energ}y_{i}$ , a binary variable indicating whether households choose clean energy, and the energy consumption intensity of household i . ${\text{NRCMS}}_{i}$ is the variable of interest, representing whether a farmer household lives in the NRCMS. ${\text{control}}_{i}$ is a vector of control variables including the individual, household and area characteristics. Furthermore, $\beta_{0}$ is the intercept term. $\beta_{1}$ is the coefficient of the impact of participation on whether a rural household consumes clean energy, if it is statistically significant, it indicates that the NRCMS is conducive to promoting clean energy consumption by rural households. $\gamma_{i}$ denotes village fixed effects, which are mainly used to exclude bias from omitted variables that change with the village, such as the level of village infrastructure. $\sigma_{t}$ denotes time-fixed effects, which are mainly used to control for influences at the time level that do not vary with the individual, such as adjustments in national macro policies. $\epsilon_{\mathrm{it}}$ is independent identically distributed random error terms, capturing some other factors that may affect the energy choice for households.

#### 4.3.2 Instrumental variable model

While our analysis employs two-period panel data, it may still suffer from omitted variable bias. Moreover, endogeneity issues arising from reverse causality could exist^4^. To address these issues, this paper employs the instrumental variable method. This involves identifying a variable that is correlated with the independent variable but unrelated to the omitted variables or the error term of the model, serving as an instrumental variable for estimation. This helps to eliminate the influence of omitted variables and reverse causality on the estimation results, enhancing the validity and credibility of the analysis. Instrumental variables should satisfy two criteria: relevance to household participation and exogeneity. In this study, we use the proportion of urban residents participating in basic medical insurance as an instrumental variable for household participation in the NRCMS. This choice is based on the premise that the participation rate in urban basic medical insurance reflects the regional promotion of basic medical insurance and the quality of medical infrastructure, which in turn affects the likelihood of rural households’ participation. Simultaneously, there is no direct correlation between urban basic medical insurance participation and rural household clean energy consumption, indicating that it does not directly influence clean energy consumption decisions. Consequently, the number of urban basic medical insurance participants meets the exclusionary and exogenous assumptions of instrumental variables and can be used to estimate the true impact of the NRCMS on clean energy consumption.

$${\text{NRCMS}}_{i}=\nu_{0}+\nu_{1}{\mathrm{IV}}_{i}+\nu_{2}{\text{Control}}_{i}+\gamma_{i}+\sigma_{t}+\rho_{\mathrm{it}}$$ (2)

$$\text{Energ}y_{i}={\nu_{0}}^{\prime }+{\nu_{1}}^{\prime }{\hat{\text{NRCMS}}}_{i}+{\nu_{2}}^{\prime }{\text{Control}}_{i}+{\gamma_{i}}^{\prime }+{\sigma_{t}}^{\prime }+{\rho_{\mathrm{it}}}^{\prime }$$ (3)

The one-stage equation is shown in Equation 2, IV is the instrumental variable of family participation; the second-stage equation is shown in Equation 3; $\rho_{\mathrm{it}}\;$ it is a random disturbance term. All other variables are consistent with Equation 1.

## 5 Empirical results and discussions

### 5.1 Empirical results

Table 2 displays the benchmark results of the NRCMS on clean energy consumption based on Equation 1. To assess the impact of participation in the NRCMS on the clean energy consumption transition of rural households, the entire estimation is performed as a stepwise regression. Among them, column (1) is the estimation results without control variables. On this basis, columns (2) to (4) present the results of estimation by including individual, household characteristic variables, and regional characteristic variables in the model. We can find that whether or not the control variables are included, the NRCMS is consistently positive and significant at the 1, 5, and 10% statistical levels, with estimated coefficients around 3%. According to the results in column (4), all else being equal, the NRCMS can promote household consumption of clean energy.

**Table 2:** The NRCMS and fuel choices: benchmark regression.

Variables	Whether to use clean fuels			
	(1)	(2)	(3)	(4)
NRCMS	0.038^***^ (0.011)	0.023^*^ (0.013)	0.028^**^ (0.012)	0.028^**^ (0.012)
Gender		−0.022^**^ (0.009)	−0.019^**^ (0.009)	−0.019^**^ (0.009)
Age		0.004^**^ (0.002)	0.000 (0.002)	0.000 (0.002)
Age^2^		−0.000^***^ (0.000)	−0.000 (0.000)	−0.000 (0.000)
Education		0.035^***^ (0.004)	0.027^***^ (0.004)	0.028^***^ (0.004)
Health		−0.003 (0.004)	−0.001 (0.004)	−0.001 (0.004)
Pension insurance		0.038^***^ (0.010)	0.030^***^ (0.010)	0.029^***^ (0.010)
Business insurance		0.055^***^ (0.011)	0.036^***^ (0.011)	0.036^***^ (0.011)
Income			0.033^***^ (0.005)	0.033^***^ (0.005)
Family size			−0.017^***^ (0.003)	−0.017^***^ (0.003)
Fuel expenditure			0.022^***^ (0.003)	0.022^***^ (0.003)
fixed assets			−0.001 (0.001)	−0.001 (0.001)
Dependency ratio			−0.063^***^ (0.019)	−0.062^***^ (0.019)
Regional economy				−0.285^***^ (0.107)
Nh				0.122 (0.142)
Constant	0.538^***^ (0.010)	0.429^***^ (0.046)	0.267^***^ (0.071)	2.491^**^ (1.255)
Time-fixed effect	Yes	Yes	Yes	Yes
Village-fixed effect	Yes	Yes	Yes	Yes
Sample size	8,977	8,504	8,427	8,427
*R* ^2^	0.385	0.401	0.415	0.416

***, **, and * denote significance at the 1, 5, and 10% levels; robust standard errors clustered at the household level are presented in parentheses.

### 5.2 Endogeneity discussion

The results are shown in Table 3, According to the regression results of the first stage of instrumental variables, which indicates that the original hypothesis of “insufficient identification of instrumental variables” is significantly rejected at the 1% level, and the Cragg-Donald Wald F-statistic is 16.38 at the 10% threshold, which suggests that the instrumental variables selected in this study are not weakly instrumental. After adding instrumental variables, it is found that the probability of households with The NRCMS consuming cleaner energy than other households is elevated compared to the previous one, specifically from 0.028 to 0.499, which suggests that the instrumental variables selected in this study well handle the benchmark regression of the endogeneity problem and highlights the importance of participation in the NRCMS for rural households’ clean energy consumption.

**Table 3:** The NRCMS and fuel choices: endogeneity discussion.

Variables	Instrumental variables for urban participation rate		Heteroskedasticity-Based Instrumental Variables	
	The first stage	The second stage	The first stage	The second stage
Instrumental Variables for Urban participation rate	2.110^***^ (0.472)			
NRCMS		0.499^*^ (0.287)		
Heteroskedasticity-Based Instrumental Variables			4.616^***^ (0.200)	
NRCMS				0.539^**^ (0.234)
Control Variables	Yes	Yes	Yes	Yes
Time-fixed effect	Yes	Yes	Yes	Yes
Village-fixed effect	Yes	Yes	Yes	Yes
Kleibergen-Paaprk LM statistic	20.141^***^		66.460^***^	
Cragg-Donald Wald F statistic	18.219		26.593	
Sample size	8,427	8,427	8,427	8,427

***, **, and * denote significance at the 1, 5, and 10% levels; robust standard errors clustered at the household level are presented in parentheses, the control variables are the same as in Table 2.

To further address concerns regarding the validity of the instrumental variable, this study follows Lewbel (35) and employs a heteroskedasticity-based instrumental variable (HBIV) approach as an additional identification strategy. The Lewbel method exploits the presence of heteroskedasticity in the data to generate internal instruments from model residuals, thereby avoiding reliance on external instrumental variables and their associated exclusion restrictions. This approach has been widely adopted in empirical studies when suitable external instruments are difficult to justify. The estimation results are reported in Table 3. Consistent with the benchmark IV estimates, the coefficient of NRCMS participation remains positive and statistically significant. The magnitude of the estimated effect is also comparable to the baseline results. These findings provide additional evidence that the positive impact of the NRCMS on rural household clean energy consumption is unlikely to be driven by endogeneity bias or the specific choice of instrumental variables.

### 5.3 Robustness checks

There are four approaches for robustness testing to confirm the robustness of the findings.

First, to mitigate estimation bias caused by sample self-selection, we employ the Propensity Score Matching (PSM) method as a robustness check. Specifically, we use whether a household participates in the New Rural Cooperative Medical Scheme (NRCMS) as the treatment variable, assigning households that participate in the NRCMS to the treatment group and those that do not to the control group, thereby establishing a reasonable counterfactual framework for the robustness test of the benchmark regression results. We select treatment and control group samples by adjusting different PSM matching ratios. The results are shown in Table 4. It is not difficult to see that regardless of the matching ratio, the regression results are consistent with those of the benchmark model.

**Table 4:** The NRCMS and fuel choices robustness checks: replacement model.

Matching method	Treatment group	Control group	ATT	Std. error
Nearest neighbor matching (1:3 matching)	0.648	0.509	0.139^***^	0.017
Nearest neighbor matching (1:5 matching)	0.648	0.506	0.142^***^	0.017

*** denote significance at the 1% level, respectively; the standard errors are those obtained by the self-help method and repeated 500 times.

Second, we excluded the samples of older population farmers. It is important to consider that older population farmers may not be significantly involved in household energy collection activities. Also, the trend of an aging population can lead to a higher proportion of household expenditure being allocated to healthcare costs, potentially at the expense of energy consumption. To avoid any potential bias in the results that may arise from the inclusion of older participants, the study excludes house-holds with older population farmers as the head of the family for a retest of the analysis. This approach is informed by the research conducted by Ren et al. (36). The findings, displayed in column (1) of Table 5, show that the impact of the NRCMS on clean energy consumption among rural households remains significant at the 5% statistical level after the exclusion of the older population sample. This reaffirms the robustness of the baseline regression results.

**Table 5:** The NRCMS and fuel choices robustness checks: sample reduction.

Variables	(1)	(2)
NRCMS	0.032^**^ (0.014)	0.633^*^ (0.336)
Control Variables	Yes	Yes
Time-fixed effect	Yes	Yes
Village-fixed effect	Yes	Yes
Kleibergen-Paap rk LM statistic	15.281^***^	16.381^***^
Cragg-Donald Wald F statistic	14.528	14.828
Sample size	6,745	7,208

***, **, and * denote significance at the 1, 5, and 10% levels; robust standard errors clustered at the household level are presented in parentheses, The control variables are the same as in Table 2; those presented in the table are the results of the second-stage regression with the addition of instrumental variables.

Moreover, we excluded the samples of poor health self-assessment numbers. The analysis excluded samples with poor health self-assessments to ensure the accuracy of the findings. Specifically, farmers who rated their health score of 1, were excluded from the regression model. This exclusion is justified by the consideration that farmers with very poor health self-assessments may face limitations in participating in agricultural activities or maintaining a normal lifestyle due to their health condition, which in turn could influence their energy consumption patterns. Including such individuals in the study could introduce bias into the results, potentially compromising the study’s accuracy and reliability. The adjusted regression results, which exclude farmers with poor health self-assessments, are presented in column (2) of Table 5. These results continue to demonstrate the robustness of the findings.

Finally, Given that the dependent variable, clean energy consumption, is defined as a binary variable, the baseline regression employs a Linear Probability Model (LPM), which is widely used in applied economics due to its straightforward interpretation and convenience in incorporating fixed effects. To examine whether the benchmark results are sensitive to model specification, we further estimate Probit and Logit models. The results are reported in Table 6. Consistent with the baseline estimates, the coefficient of NRCMS participation remains positive and statistically significant across both nonlinear specifications. In addition, the magnitude and direction of the marginal effects are broadly comparable to those obtained from the baseline model. These findings suggest that the positive effect of NRCMS participation on rural household clean energy consumption is robust to alternative estimation methods and is unlikely to be driven by model selection.

**Table 6:** The NRCMS and fuel choices robustness checks: different model.

Variables	Logit	Probit
NRCMS	0.260^***^ (0.095)	0.168^***^ (0.047)
Control Variables	Yes	Yes
Time-fixed effect	Yes	Yes
Village-fixed effect	Yes	Yes
Sample size	7,469	7,469
Log likelihood	−3438.064	3435.676

*** denote significance at the 1% level; robust standard errors clustered at the household level are presented in parentheses, the control variables are the same as in Table 2.

### 5.4 Analysis of the mechanisms by which social security influences clean energy consumption in rural households

#### 5.4.1 Wealth effect

The wealth effect mainly consists of easing household medical burdens and reducing precautionary savings. Among them, regarding the measurement of medical burden, this paper draws on Yu et al. (37), and this paper adopts the share of household medical consumption expenditure in total household income to characterize it; regarding household precautionary savings, it generally refers to the savings made by risk-averse consumers to prevent future uncertainties that may lead to a sharp decline in consumption level (38). Some studies even suggest that precautionary savings explain at least about 20 to 30% of the per capita financial property accumulation of urban and rural residents in China (39). Therefore, this paper uses the household savings rate to approximate the precautionary savings of households.

The regression results are shown in Table 6. Column (1) shows the results of the mechanism test of medical burden, and the effect of enrollment on the medical burden of households is significantly negative, indicating that enrollment can reduce the medical burden of households under all other conditions, and participation in the NRCMS can alleviate the medical burden of households and enhance the optional set of household consumption, and energy consumption is an important part of household consumption, so enrolled households will tend to choose more environmentally friendly and convenient cleaner energy on their energy consumption. Will tend to choose cleaner energy that is more environmentally friendly and convenient. Column (2) shows that the precautionary savings channel is not statistically significant. One possible explanation is that precautionary savings among rural households are driven by multiple sources of uncertainty rather than medical risks alone. In addition to health expenditures, households often face substantial future financial obligations related to housing construction, children’s education, old-age support, and marriage-related expenditures. Under such circumstances, a reduction in medical uncertainty may not be sufficient to alter saving behavior significantly. In rural China, households face multiple high-priority expenditures—such as marriage, housing, and education—which may crowd out energy-related investments (40). In addition, clean energy adoption is often characterized by a degree of postponability and does not constitute an urgent or irreversible investment. As suggested by consumption commitment theory (25), households tend to prioritize expenditures with stronger commitment features, while more flexible consumption items, such as energy choices, are more likely to be delayed. Therefore, although the NRCMS reduces uncertainty and precautionary savings, the reallocation of resources toward clean energy is not automatic, which may explain the insignificant empirical results.

This result highlights an important boundary condition of the precautionary-saving mechanism. The mechanism is more likely to operate when future expenditure pressures are relatively limited or when medical risk constitutes the dominant source of uncertainty. In contrast, the medical-burden channel directly relaxes current budget constraints and therefore exerts a more immediate and robust influence on household energy choices.

#### 5.4.2 Health effect

Due to data limitations, the mechanism analysis primarily focuses on the health-cognition pathway. Although previous studies suggest that health insurance may influence household behavior through improvements in health status, the available CFPS data do not allow us to accurately identify the long-term behavioral consequences of health improvement. Therefore, our mechanism test provides evidence mainly for the health-cognition channel. The health effect is a measure of the importance respondents attach to their health. According to Grossman's (41) health capital theory, individuals invest in their health—including through healthcare expenditures—to increase healthy time. Consequently, household monetary spending on health can be considered a revealed preference for health and an indicator of health awareness. Based on this theoretical framework, this paper uses household health expenditure to measure respondents’ health importance. Household health expenditure data are derived from the total annual household spending on medical care (including health supplements, health devices, etc.) as reported in the questionnaire. A higher level of such expenditure indicates that respondents and their families place greater importance on their health. The regression results of the model are presented in column (3) of Table 7, indicating that, all else being equal, the NRCMS increases respondents’ importance attached to their health.

**Table 7:** The NRCMS and fuel choices: mechanism test.

Variables	(1)	(2)	(3)
	Medical burden	Precautionary savings	Health effect
NRCMS	−3.762^*^ (1.945)	0.051 (0.379)	1.318^***^ (0.251)
Control Variables	Yes	Yes	Yes
Time-fixed effect	Yes	Yes	Yes
Village-fixed effect	Yes	Yes	Yes
Kleibergen-Paap rk LM statistic	20.950^***^	19.144^***^	32.141^***^
Cragg-Donald Wald F statistic	19.258	17.369	20.378
Sample size	8,402	8,403	8,260

*** and * denote significance at the 1 and 10% levels; robust standard errors clustered at the household level are presented in parentheses, the control variables are the same as in Table 2; those presented in the table are the results of the second-stage regression with the addition of instrumental variables.

### 5.5 Heterogeneity analysis

#### 5.5.1 Heterogeneous effect based on the potential future household expenditure pressure

In traditional Chinese culture, the marriage is considered a paramount family affair, with parental investment in a child’s wedding often seen as a parental obligation. This is particularly pronounced in some rural areas of China, where the cost of a son’s wedding can consume the average family’s accumulated wealth over a decade. This exorbitant expenditure has given rise to a form of intergenerational exploitation within the family. The gender imbalance in China has led to a tightened marriage market (40), prompting parents to raise their household savings and bride price to enhance their son’s chances of securing a marriage partner (42). Additionally, parents often face the burden of substantial dowry payments, which further exacerbates the economic pressure on families.

We posit that in rural households, the number of unmarried children is directly proportional to the potential future household expenditure pressure. While the NRCMS can alleviate some of this financial burden, it may not be sufficient in the face of dowry demands, making it challenging to directly incentivize rural families to adopt more expensive, cleaner energy sources. To account for this, we categorize households based on the number of unmarried individuals. We define households with a share of unmarried members above the median as those with a higher proportion of unmarried individuals, and those with a share below or equal to the median as those with a lower proportion. Our regression results, presented in Table 8, indicate that the NRCMS impact on clean energy consumption varies significantly across these subgroups. Households with a lower proportion of unmarried members are more likely to experience the incentivizing effect of the NRCMS and are thus more cautious in their selection of clean energy sources.

**Table 8:** The NRCMS and fuel choices: household expenditure pressure heterogeneity.

Variables	Whether to use clean fuels	
	Low expenditure pressure	High expenditure pressure
NRCMS	0.718^*^ (0.426)	0.326 (0.312)
Control Variables	Yes	Yes
Time-fixed effect	Yes	Yes
Village-fixed effect	Yes	Yes
Kleibergen-Paap rk LM statistic	12.015^***^	13.195^***^
Cragg-Donald Wald F statistic	11.841	16.118
Sample size	4,209	4,146

*** and * denote significance at the 1 and 10% levels; robust standard errors clustered at the household level are presented in parentheses, The control variables are the same as in Table 2; those presented in the table are the results of the second-stage regression with the addition of instrumental variables.

#### 5.5.2 Heterogeneity effects based on household labor allocation

In recent years, as China has experienced rapid economic growth, it has witnessed a massive rural-to-urban migration. However, the dualistic barriers within the urban–rural social security system prevent migrant workers from fully enjoying the same social welfare benefits as urban residents (43), contributing to social inequality. The NRCMS, according to the Push and Pull Theory, has a positive “push” effect, encouraging laborers to seek employment outside their rural areas. Conversely, urban residents’ medical insurance exerts a “pull-back” effect, restricting the population’s mobility and ability to work outside their hometowns. Moreover, the partial reimbursement of hospitalization costs has a limited effect on reducing the healthcare burden of rural residents if they are not reimbursed from other sources (44). Currently, farmers encounter cumbersome procedures and multiple barriers when seeking local and cross-location reimbursement for the NRCMS. Against this backdrop, this paper argues that due to the urban–rural health insurance interface, migrant workers may have a lower perception of the welfare effect of the NRCMS and expect it to provide insufficient protection. This perception makes it difficult to incentivize them to consume clean energy. Also, long-term migration reduces the amount of time household members spend at home, thereby weakening incentives to improve indoor living environments through clean energy adoption. Consequently, the marginal effect of NRCMS participation on energy choices may be smaller for migrant-worker households.

To validate the above, we assign a value of 1 to households with an outworker ratio equal to or greater than the median of the sample, and a value of 0 to households with a ratio below the median, based on the proportion of outworkers among the family members of the farm household. The study’s findings reveal that only the low-out-worker sample group exhibits significance at the 10% statistical level. This suggests that the NRCMS is insufficient in safeguarding the outworking group from the uncertainties they face, thus hindering their consumption of clean energy (see Table 9).

**Table 9:** The NRCMS and fuel choices: labor allocation heterogeneity.

Variables	Whether to use clean fuels	
	Large share	Small share
NRCMS	0.105 (0.432)	0.602^*^ (0.360)
Control Variables	Yes	Yes
Time-fixed effect	Yes	Yes
Village-fixed effect	Yes	Yes
Kleibergen-Paap rk LM statistic	8.946^***^	16.086^***^
Cragg-Donald Wald F statistic	8.120	13.169
Sample size	4,083	4,255

*** and * denote significance at the 1 and 10% levels; robust standard errors clustered at the household level are presented in parentheses, The control variables are the same as in Table 2; those presented in the table are the results of the second-stage regression with the addition of instrumental variables.

#### 5.5.3 Heterogeneity effects based on regional healthcare resource endowment

Under the conditions of China’s ever-improving social security system, the coverage of the New Farmers’ Cooperative has been increasing. However, China has long had a pattern of uneven distribution of medical resources, with an insufficient total number of large, high-quality hospitals and an unbalanced structural distribution, especially in some of the developed regions. This has created a contradiction between the uneven distribution of medical resources and people’s growing health needs (45), and some residents will choose to seek medical treatment across regions to obtain good medical services, according to the statistics, average, there are about 125 million cross-provincial migrant populations each year with the demand for medical settlements. However, China’s policy requires all patients seeking medical treatment across provinces to obtain prior authorization for direct settlement and to pay for their medical expenses according to the list of medical insurance in the region where they seek treatment and the reimbursement rates in the participating regions. This can, to some extent, lead to differences in access to medical care for patients in the same participating region due to differences in the health insurance lists of the regions where they seek care (46).

In addition, some scholars have found that in some regions, in order to prevent the outflow of patients, regional healthcare departments have even reduced the payment ratio of inter-regional medical care for some programs (47). Based on this, this paper uses the sum of the number of practicing physicians per 1,000 people and the number of beds per 1,000 people in the region to measure the resource endowment status of the region and incorporates the interaction term between the region’s healthcare resource endowment and the core explanatory variables into the model to examine the heterogeneity of healthcare resource endowment. The regression results are shown in Table 10, and it is not difficult to find that the cross-multiplication term of the two has a significant positive effect on rural household clean energy, that is, In regions with better medical resource endowment, households can more easily translate insurance coverage into actual healthcare utilization. As a result, the economic and health benefits of the NRCMS are more likely to materialize, strengthening its influence on household energy choices. The coefficient of the cross-multiplier term is 0.133, and the average interaction effect of the cross-multiplier term obtained after the transformation is 0.016, i.e., for every unit of improvement in regional medical resource endowment, the promotion effect of participation in the cooperative on the clean energy consumption of rural households will increase by 0.016 units.

**Table 10:** The NRCMS and fuel choices: healthcare resource endowment heterogeneity.

Variables	Whether to use clean fuels	
	(1)	(2)
NRCMS	0.499^*^ (0.287)	−1.211^*^ (0.710)
NRCMS*Healthcare resource endowment		0.133^*^ (0.076)
Healthcare resource endowment		0.037 (0.060)
Control Variables	Yes	Yes
Time-fixed effect	Yes	Yes
Village-fixed effect	Yes	Yes
Kleibergen-Paap rk LM statistic	20.141^***^	80.055^***^
Cragg-Donald Wald F statistic	18.219	119.010
Sample size	8,427	8,427

***, **, and * denote significance at the 1, 5, and 10% levels; robust standard errors clustered at the household level are presented in parentheses, the control variables are the same as in Table 2; those presented in the table are the results of the second-stage regression with the addition of instrumental variables. The tool variable of NRCMS*Healthcare resource endowment in column (2) is NRCMS*Healthcare*IV.

## 6 Discussion

### 6.1 Results discussion

This study examined the impact of participation in the NRCMS on rural household clean energy consumption using panel data from the China Family Panel Studies (CFPS) for 2018 and 2020. We found that NRCMS participation significantly increased the probability of adopting clean energy by approximately 2.8 percentage points, with the effect operating primarily through the wealth effect (reducing medical burden), while the precautionary savings channel was not statistically significant. The health effect (enhancing health awareness) also played an important role.

Consistent with previous research (26), our findings indicate that the NRCMS relaxes household budget constraints by reducing out-of-pocket medical expenditures, thereby freeing disposable income for clean energy investments. However, contrary to studies suggesting a precautionary savings mechanism (27), we found no significant support for this channel. This discrepancy may be explained by competing household expenditure demands in rural China—such as bride price, housing, and education (40)—which crowd out clean energy investments due to their non-postponable nature (25). Our health effect findings support Grossman’s (41) health capital theory and align with Alem et al. (30), who emphasized the role of health awareness in promoting clean energy use. To our knowledge, this study provides one of the first attempts to link the health-education function of the NRCMS to household energy choices.

The heterogeneity analysis revealed several important patterns. First, households with fewer unmarried members were more responsive to the NRCMS. This is likely because marriage-related expenditures (e.g., bride price) create strong saving motives that crowd out clean energy investment (48), consistent with the competitive saving motive documented by Wei and Zhang (40). Second, households with fewer migrant workers also showed stronger effects, suggesting that the complexity of cross-provincial medical reimbursement reduces migrant workers’ perceived value of the NRCMS (44). Third, for every one-unit increase in regional medical resource endowment, the promoting effect of NRCMS participation on clean energy consumption increased by 0.016 units. This finding aligns with Zhang et al. (45), who showed that unequal healthcare access affects household behavior, and implies that better local medical services reduce the need for cross-regional travel, thereby amplifying the spillover effect.

This study contributes theoretically by incorporating health insurance into rural energy transition analysis and revealing heterogeneous wealth and health pathways. Practically, it offers policy insights for developing countries to promote clean energy transition by optimizing reimbursement rates, addressing unmarried and migrant-worker households, and improving regional medical resource endowments.

### 6.2 Policy implications

The above findings have significant policy implications for China and other developing countries on how to improve the health insurance to promote rural energy transition. First, policymakers should strengthen the NRCMS by increasing financial support, raising reimbursement rates for outpatient and inpatient care, and expanding coverage to stabilize household expectations. Second, a unified national health insurance system should be established, moving from municipal to provincial coordination and simplifying cross-provincial reimbursement to better serve migrant workers. Third, complementary measures are needed: for households with many unmarried members, guide rational views on bride price and provide low-interest loans for clean energy equipment; for migrant-worker households, improve reimbursement portability and increase ratios; for resource-poor regions, invest in healthcare infrastructure and raise local reimbursement rates to reduce cross-regional travel and enhance the NRCMS spillover effect on clean energy.

### 6.3 Limitations and future research directions

Limited by data availability and the scope of the analysis, several limitations in this study should be acknowledged and can be addressed in future research. Specifically: (1) This study measures clean energy consumption using households’ primary cooking fuel. Because the CFPS only provides information on the primary fuel type and does not report the quantity, share, or composition of different energy sources consumed by households, it is not possible to accurately characterize fuel-stacking behavior at the national level. Accordingly, the focus of this study is on households’ primary energy choices rather than their complete energy consumption structure. Future research could employ more detailed household energy datasets to examine how health insurance influences energy portfolios, clean energy intensity, and fuel-stacking behavior. (2) This study primarily focuses on the wealth and health mechanisms through which the NRCMS affects household clean energy consumption, with the precautionary savings channel found to be insignificant. Future studies could explore alternative behavioral mechanisms, such as changes in time preferences, risk attitudes, or social norms induced by health insurance, to better understand why some pathways dominate over others. (3) The heterogeneity analysis reveals that household composition (e.g., number of unmarried members) and labor allocation (e.g., share of migrant workers) moderate the NRCMS effect. Future studies could further investigate how other demographic and socio-cultural factors—such as intergenerational co-residence, gender dynamics within the household, or local marriage market conditions—shape the relationship between health insurance and energy choices. (4) Other potentially important determinants of household clean energy adoption, such as access to energy infrastructure, relative energy prices, seasonal variations, and intra-household decision-making power, deserve further quantitative investigation in future work, ideally integrating survey data with administrative or geospatial data.

Despite these limitations, this study provides new evidence on how social protection policies influence household behavior beyond healthcare. As China and other developing countries expand health insurance coverage while facing urgent needs for rural energy transition, our findings suggest that improving medical insurance schemes may serve as an important policy tool for promoting energy consumption. Future studies may explore potential coordination mechanisms between health insurance policies and clean-energy promotion programs.

## Data Availability

Publicly available datasets were analyzed in this study. This data can be found at: http://www.isss.pku.edu.cn/cfps/.

## Footnotes

1 The policy background of NRCMS has been presented in the Appendix.

2 Source document for sample sampling design principles: https://www.isss.pku.edu.cn/cfps/docs/20200520161539050175.pdf

3 Since the CFPS data does not publicize county-level codes, this paper uses provincial-level data when matching macroeconomic data.

4 If farmers’ prolonged use of non-clean energy results in health risks, they might be more likely to join the NRCMS to mitigate the financial burden of medical expenses. Conversely, farmers who consume clean energy may be inherently more health-conscious and thus more motivated to purchase NRCMS to secure medical insurance and social benefits.

## Appendix

### The policy background of NRCMS

The NRCMS is a health insurance program introduced by the Chinese government to benefit rural residents. Initially launched as a pilot project in 310 villages across the country in 2003, expanded to 617 villages in 2005, and fully implemented by 2007. Its primary goal is to alleviate the financial burden of rural residents in case of illness. The system is rooted at the village level and operates through the voluntary enrollment of farmers, government financial subsidies, and the establishment of community health service centers. Together, these components form a novel rural medical insurance system that blends the principles of insurance with mutual aid. The key characteristics of the NRCMS are as follows: (1) it is tailored for rural households; (2) the fundamental unit of participation is the family, not the individual; and (3) contributions to the NRCMS comprise two main elements: state financial subsidies and the contributions of participating families. The government’s financial subsidy is sourced from central, provincial, and county-level subsidies, with premiums set at a uniform rate within the same region, and individual contributions calculated as 0.57 percent of the urban and rural residents’ per capita disposable income from the previous two years. If individual contributions fall short of the standards set by the state and province, the established state and provincial standards apply, acknowledging the varying economic levels across regions and resulting in different criteria for state financial subsidies and individual farmer contributions.^5^
